# Gasification of sewage sludge within a circular economy perspective: a Polish case study

**DOI:** 10.1007/s11356-019-05897-2

**Published:** 2019-07-22

**Authors:** Sebastian Werle, Szymon Sobek

**Affiliations:** Institute of Thermal Technology, Silesian University of Technology, 44-100 Gliwice, Poland

**Keywords:** Waste biomass, Bioeconomy, Thermal methods, Phosphorus, Extraction, Fertilizer, Poland

## Abstract

Sewage sludge (SS) is a by-product of wastewater treatment plant (WWTP) operation. Due to fast rates of urbanization and industrialization, and rapid population growth, the world community faces a serious challenge associated with its disposal. There is an urgent need to explore low cost, energy efficient, and sustainable solutions for the treatment, management, and future utilization of SS. Thermal conversion of SS is considered the most promising alternative for sustainable SS management. Among three main thermochemical processes, it seems that gasification (GAS) of SS has the most advantages. The aim of this paper is a presentation of the gasification process as a sustainable method of SS management that takes into account the idea of a circular economy (CE). Gaseous fuel production, phosphorus recovery potential, and solid adsorbent production during the gasification process are analyzed and discussed. Result of this study shows that the lower heating value (LHV) of the gas from SS GAS process is up to 5 MJ/m^3^_n_ and it can be effectively utilize in an internal combustion engines. The analysis proved that solid fraction after the SS GAS process can be treated as a valuable phosphorus source and perspective adsorbent materials. The amount of P_2_O_5_ in this material was equal to 22.06%. It is similar to natural phosphate rocks (28.05%). The maximum of the adsorption capacity of the phenol was comparable with commercial activated carbon (CAC): 42.22 mg/g for solid fraction after SS GAS and 49.72 mg/g for CAC.

**Figure:**
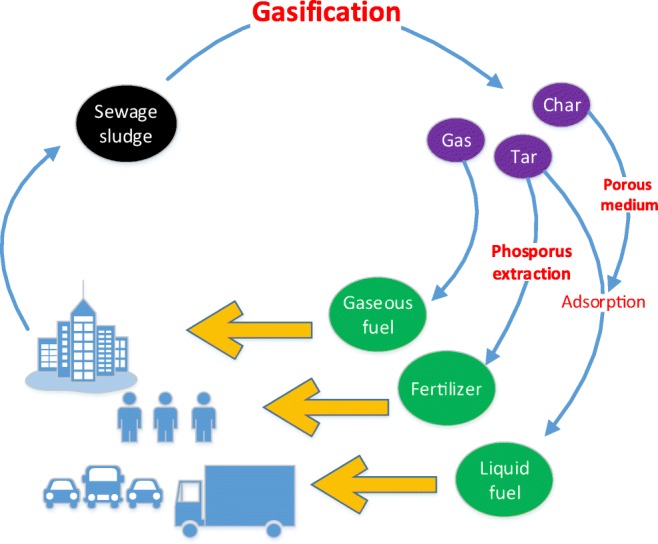
Graphical abstract

Graphical abstract

## Introduction

In 2016, almost 80% of world’s final consumption of the energy is still provided by fossil fuel utilization. Though, this is continuously being diminished by the rising growth rate in modern renewables. Increases in renewable energy deployment continued in 2017, especially in the power sector, thanks to increasing access to finance, global concerns about energy security, human health and the environment, growing energy demand in young and developing economies, an urgent need for emission-free electric energy and clean cooking facilities, dedicated policy initiatives, and support for ambitious targets (Eckhart et al. 2018). With continuous economic and population growth, global energy demand is rising, while available fossil fuel resources are slowly approaching exhaustion. Throughout the previous 50 years, the total population doubled more quickly than at any other time in recent memory, and more quickly than it is anticipated to develop later on. In 1950, the world had 2.5 billion individuals; and in 2005, the world had 6.5 billion individuals. According to World Population Prospects (2017), by 2050, this number could ascend to in excess of 9 billion or more, and will exceed 11 billion by 2100. The rapidly rising demand for energy has encouraged the alarmingly intensive exploitation of the natural environment in search of the new renewable energy sources. This exploitation also have significantly influenced the generation of municipal and industrial waste around the globe (Matsunaga and Themelis 2002). Similarly, Hoornweg and Bhada-Tata (2012) posit that rising urban populations have increased solid waste generation by tenfold around the globe. Moreover, global estimates indicate solid waste generation will double from 3.5 million to 6 million tons/day by 2025 exceeding environmental pollutants and greenhouse gases (GHGs). In corroboration Werle (2015) asserts that rising solid waste generation prompted by rising wealth and population dynamics will increase pressure on current waste management (WM) systems. Furthermore, this will result in significant socioeconomic, environmental, technological, and geopolitical implications globally.

Sewage sludge (SS) is an one of example of solid waste and, simultaneously, is a major by-product of wastewater treatment plant (WWTP) operation. Due to increasing urbanization and industrialization, caused by rapid population growth, SS disposal is one of the key issues of today’s waste economy. On a global scale, daily production of sludge varies widely from 35 to 85 g dry matter (dm) per capita per day (IWA 2019). Further increases have been projected due to aforementioned population growth. Despite the SS disgrace, its growing production and the enormous cost of disposal has led engineers to consider its energy potential and propose it as a potential new renewable. Many countries (e.g., Slovakia, Poland and China) are considering or are already implementing thermal methods of SS conversion (Hronocova et al. 2017; Syed-Shatir et al. 2017; Haibo et al. 2019). In the European Union (EU), the SS problem is being tackled by the general Directives, indicators, and national legislative requirements.

### Regulations concerning SS disposal

Currently, SS disposal is regulated by the Council Directive 86/278/EEC, established by the EU in 1986, the so-called “Sludge Directive.” The next document connected with SS disposal is Directive 2000/60/EC (Water Framework Directive - WFD). This policy, introduced by the European Parliament in the year 2000, describes regulations in reference to the water policy and defines SS as a product of sewage treatment and no longer as a waste material. The operational document of the WFD is the Directive 91/271/EEC from 1991. It defines possible ways for municipal SS treatment and obliges cities to monitor and report final methods of municipal SS disposal. This approach is focused mainly on the reuse of SS as a valuable material. The final effect of the implementation of Directive 91/271/EEC, until 2015, was the increased production of SS. However, it opened the door for alternative re-use methods of SS as a valuable material. Another EU document which regulates storage issues related to SS is Directive 99/31/EC from the 1999 Landfill Directive. Recycling of SS is the subject of the Directive 2008/98/EC. This document takes the position that the key priority is the prevention of SS production. The next priority is the preparation of SS for recycling and recovery. It is proposed that this recovery should be mainly related to energy production from the sludge in the Waste-to-Energy (WtE) model.

The idea of WtE has grown fundamentally in recent decades. With improvements starting during the 1990s, today’s WtE systems have been incredibly modernized and increasingly organized. The types of feedstock utilized have likewise expanded. SS is also recognized as a valuable feedstock for this purpose. Sludge into energy (StE) processes have been treated as one of the best methods to handle the problem of the growing amount of SS associated with the increase in the number of WWTP in the world (Chen et al. 2016; Ferrasse et al. 2003; Samolada and Zabaniotou 2014). Thermo-chemical processes offer not only real amount decrement, but are also effective for pathogen management and the potential valorization of energy-rich content (Jiang et al. 2016). In practice, technological strategies may also allow for the recuperation of valuable nutrients and metals (Bridle and Pritchard 2004; Mulchandani and Westerhoff 2016; Donatello and Cheeseman 2013).

Pyrolysis, gasification, and incineration are the most popular conventional paths of thermo-chemical treatment. Regarding the technical and environmental aspects of the sewage-to-energy concept within the CE (circular economy) (i.e., knowledge of technology, technological complexity, and hazardous emissions), gasification seems to be the most prominent conversion methods within the CE concept (Muzyka et al. 2015).

### Gasification as a SS conversion method

The gasification process is the incomplete oxidation of biodegradable material in an oxidant-restricted atmosphere. The process exists in the gasification reactor, called the gasifiers. The reactors can be divided into three main types (Gorazda et al. 2017; Werle 2014): fixed bed (FBG), fluidized bed (FlBG), and entrained bed gasifiers (EBG). The main purpose of this process is flammable gas production (e.g., H_2_, CO, and CH_4_, known as “gasification gas”). Gasification also produces a solid fraction, which is a carbon rich material (Skorek-Osikowska et al. 2017). Gasification gas is commonly used as a fuel. It can be utilized in mechanical or electrical power generation processes. Sometimes it can be utilized as a second fuel in diesel engines working in “dual fuel” operations (Oh et al. 2019). These power systems based on gasification are technologically mature.

In Europe, there are many gasification plants. The most popular and widely known is in 8 MW’s power Güssing (AUT), built in 2001. Other examples include plants in Kokemäki (FIN), Skive (DK), or Spiez (SUI) (Uchman and Werle 2016). Generally, it should be said that gasification systems are usually realized on a scale not much larger than combustion systems (Pinto et al. 2007).

Gasification is tolerant of diverse feedstocks, especially contaminated. Taking into consideration that gasification is characterized by a reductive environment, the total volume of produced gas is lower than the volume of exhaust from the combustion process. Consequently, sulphur present in gasified material is transformed to H_2_S, nitrogen to NH_3_, chlorides to HCl. The formation of dioxins, SO_2_, and NO_x_ is prevented, and gas cleaning installation is smaller and less expensive compared to classic combustion (Werle 2013). This feature is very profitable, taking into consideration the possible use of such an installation on the site of a WWTP. In such context, it is assumed that the sewage sludge can be a good fuel for this application. First trials of the SS gasification were focused on the proving that there is possibility of co-gasification of the SS with conventional biomass (e.g., wood) and waste (e.g., solid recovered fuels). The results obtained by Seggiani et al. (2012) showed that it is possible to co-gasify SS (70%) with wood pellets (30%) in FBG unit. It was concluded that, due to high ash content and low ash fusion temperatures, a slagging problem was found. This phenomenon makes the gasification process unstable. Vonk et al. (2019) performed a study on co-gasification of wood with solid recovered fuels, waste tires, plastics, and SS. Experiments showed that a mixture of dried SS (20%) and waste wood (80%) resulted in good efficiency in the gasifier, although a lower hydrogen content was obtained in this work. This can be explained by the high iron load in the SS, reaching up to 7.7% (mass fraction). A study performed by Thomsen et al. (2017) concluded that LT-CFB (low-temperature circulating fluidized bed) gasification and co-gasification is a very effective method to manage SS.

The positive experience with SS co-gasification has provided the basis for the development of the gasification process of the sludge themselves. Researchers pay attention to the problem of pollution in SS.

During SS gasification, the occurrence of inorganic and organic contamination, including waste by-products (ash and tar), is also important (Werle and Dudziak 2014). In that context, new procedures are continuously generated (Werle and Dudziak 2013; Werle et al. 2016). For example, in non-treated (or raw) SS, as well as in the post-process, tars both organic (e.g., PAHs–polycyclic aromatic hydrocarbons) and inorganic substances (e.g., HM–heavy metals) were identified. In the case of ash, mainly inorganic heavy metals were detected. The inorganic and organic contamination is transported close within the system, SS gasification by-products. In order to determine contaminants, basic instrumental methods (gas chromatography and absorption spectrometry) (Werle and Dudziak 2013) can be used, as well as indirect methods like photoacoustic spectrometry or ecotoxicological analysis (Werle et al. 2016).

In recent years, it is postulated that gasification is the best way to implement the circular economy concept.

### Sludge-to-energy in the CE concept

The concept of the CE was not a recent invention. Rather, it was introduced by British economists Pearce and Turner in 1989. This term was then better described more than 20 years later by the Ellen MacArthur Foundation (Potocnik 2013). This definition stated that CE is a “restorative economy or regenerative by intention and design”.

In 2015, the EU was introduced to the CE concept. This document establishes closed-loop flow of materials, efficient use of resources and energy, prevention of waste deposition, as well as, reuse of waste, by-products, and secondary raw materials (Lozano-Lunar et al. 2019). SS, as a product of WWT, has big potential and must be recycled under the CE idea. This necessity results, on the one hand, from the formal and legal requirements related to the prohibition on SS storage, the increase in produced SS due to the growth the population, and the introduction of sustainable development principles, but—on the other hand—from the increase in awareness of the usefulness of SS as an energy raw material. The CE is right now very important to the EU agenda, so all of the EU countries should change commonly used techniques of SS utilization and move into more efficient waste treatment, embracing the CE idea.

The European Commission recently published the Document (2017), which aims to clarify the role of WtE for the CE. It states that “WtE processes can play a role in the transition to a CE, provided that the EU waste hierarchy is used as a guiding principle and that choices made do not prevent high levels of prevention, reuse, and recycling.” Recently, European policy making has taken into consideration the CE idea. The economic growth with incremental environmental issues has to be introduced. Solutions-based perspectives for achieving economic development within increasing environmental constraints have been denoted. Additionally, a number of European countries have been encouraged to indicate the CE as their political priority. Rigueiro-Rodriguez et al. (2018) proposed changes for SS applications based on Zn balance within a CE perspective. The authors stated that regulations for agriculturally used SS (e.g., fertilizers) should aim to reach mean values of Zn. The soil microbial health should be maintained. For this reason, low quality SS application should be reduced. The CE perspective for SS extends from the possibility of phosphorus recovery in WWTP (Wilfert et al. 2018) to construction industry for material for cement production (Smol et al. 2015) and as a fertilizer in acid soils (Mosquera-Losada et al. 2017).

Meeting the CE assumptions requires an urgent need to carry out essential changes in all EU member states, including Poland (Ciuła et al. 2018). Thus, SS management in Poland will be analyzed in the following sections of this paper.

### Poland—general information

The Republic of Poland is a central European nation located east of Germany on the geographical coordinates 52° N 20° E with a total land mass of 312,685 km^2^. The land mass is comprised of 2.71% water and 97.3% land with 3070 km^2^ of territorial boundaries shared by seven countries including Belarus, Czech Republic, Germany, Lithuania, Russia (Kaliningrad Region), Slovakia, and Ukraine. The landscape consists of plains with mountains along the borders in the south, which account for its temperate climate. The seasonal weather is typically characterized by moderate to severe cold, cloudy winter conditions with regular rainfall, whereas summers are mild with periodic showers and thunderstorms.

According to 2016 estimates, Poland is inhabited by 38.5 million people, comprising 96.90% Polish, 1.10 % Silesian, 0.20% German, 0.10% Ukrainian, and 1.70% other nationalities. The economy of Poland is dominated by the service sector at 55.60%, with the remaining sectors being primarily comprised of industrial manufacturing (41.10%) and commercial agriculture (3.30%). These three sectors jointly account for USD$1.005 trillion of Poland’s gross domestic product. According to the CIA World Factbook (2016), the per capita income of the average Pole is estimated at USD$26,000, which buttresses the nation’s high living standards. As was mentioned above, the increasing of living standards causes the increment of the waste (e.g., sludge) production. The same is in Poland.

### Forecasts for SS management in Poland

Based on the forecast for Poland, defined in Resolution no. 88 of the Council of Ministers of 11 August 2016 on the “National Waste Management Plan 2022,” it should be assumed that the total mass of municipal SS in 2020 will be equal to 750,000 Mg (dm).

Table 1 presents data on the disposal, management, and utilization of the total industrial and municipal SS produced from over 4255 WWTPs operating in Poland for the year 2016.

**Table 1:** Sewage sludge (SS) management in Poland for 2016 (Statistical Yearbook of the Regions)

Sewage sludge (SS) utilization	Total SS (industrial and municipal), tonnes of dry solid
Land reclamation	31,724
Compost production	32,807
Bulk storage	61,889
Landfilling	97,569
Agriculture	133,887
Thermal conversion	194,677
Other uses	394,638
Accumulated*	6,286,969

^*^Total annual SS accumulated on the WWTP on landfill areas

Taking into consideration the numbers presented in Table 1, it should be concluded that, SS is mostly utilized for agriculture, compost production, thermal conversion, bulk storage, and other industrial uses. It should be emphasized that in terms of the EU and national formal documents, this structure of SS disposal is not acceptable. The main problem is the small amount of thermally utilized sludge. Most of the accumulated SS constitute an unused source for thermal conversion use. Thermal processes can be implemented at existing heating or power plants, or can by erected as a completely new facility. Such installations can convert large amounts of SS. This is a very advantageous feature of thermal methods. Moreover, taking into consideration the properties of SS, among all thermal process gasification seems to be the best option. Gasification is a prominent technology, which fits exceptionally well into the CE concept. This process ensures complete sterilization of SS, effective mass reduction to solid fraction, and provides an opportunity to recover valuable materials.

The paper focuses on showing that gasification is an effective method of the sewage sludge management taking into account the CE idea. For this purpose, an experimental analysis of the municipal sewage sludge gasification process including the determination of the phosphorus recovery potential from the solid waste products taken from the process. Moreover, the experimental analysis of the phenol adsorption from water solution using solid waste gasification process is presented. The influence of the air ratio on the temperature distribution in the gasifier, the gaseous fuel composition, and its lower heating value are discussed. The amount of phosphorus existed in the solid waste process product is determined. The comparison to natural phosphate rock is presented. The comparison of the adsorption efficiency of phenol on solid gasification product with various normally used adsorbents is presented.

## Materials and methods

Gasification experimental setup

**Fig. 1.**
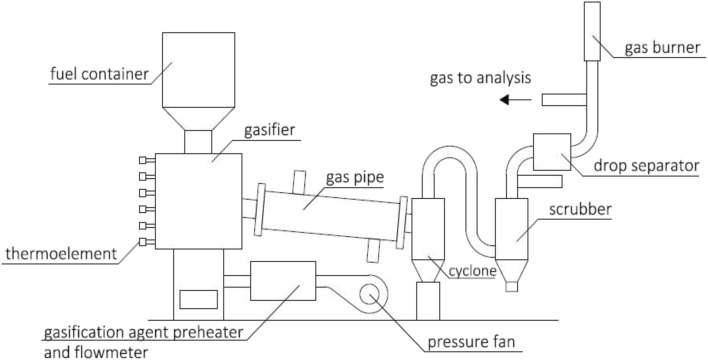
The scheme of the FBG reactor (Werle and Wilk 2011)

The SS gasification experiment was realized. For the present study, a FBG reactor was used. The whole system is presented in Fig. 1. The objective of the experiment was to determine the influence of the air ratio on the temperature conditions in the gasifier and on the properties of the achieved gaseous fuel. The process also generates the solid waste material for phosphorus recovery potential analysis and the phenol adsorption process investigation.

The key reactor element was an insulated, stainless gasifier pipe. The internal diameter of the pipe was equal to 150 mm and the total height was equal to 300 mm. SS, in the form of granules, was introduced to the reactor from the top located fuel box. The gasification agent was fed bottom up by the pressure fan. Six N-type thermoelements were used for measuring the temperature inside the reactor. All of the thermocouples were located along the vertical axis of the gasifier and were connected to the temperature recording system (Agilent Company). In addition to the temperatures in the reactor, the temperature of the gas leaving the installation was also measured. The volume flow rate of the gasification agent and the flow rate of gasification gas were measured by flow meters. Gasification gas was transported by the gas pipeline and was then cleaned by a gas cleaning installation. This consisted of a cyclone, a scrubber, and a drop separator. The main components of the gasification gas were measured online using a set of analyzers.

### Gasification experimental methodology

In Table 2, the gasification methodology has been presented.

**Table 2.** Experimental matrix of gasification process

Sewage sludge (SS)	Gasification agent	Air ratio *λ*, -	Tests
SS1(MB system) SS2 (MBC system)	Atmospheric air at ambient temperature	Adjustable from 0.12 to 0.27	(i) Fuel production (ii) P recovery (fertilizer purposes) (iii) Sorbent production

The experiments were carried out using two types of sludge. The first (SS1) from the Polish WWTP operated in mechanical-biological (MB) system, and the second (SS2) from the Polish WWTP operated in the mechanical-biological-chemical (MBC) system. Both systems included a stage of dewatering, anaerobic digestion stabilization, and mechanical drying.

### Adsorption experiment methodology

The experiment of adsorption was realized in the static environment. Erlenmeyer flasks were used. The objective of the research was the determination of the efficiency of adsorption of phenol on solid waste product which generates from the SS gasification process and comparison values of this parameter achieved using other materials.

Process parameters are displayed in Table 3.

**Table 3.** Parameters of the adsorption process

Parameter	Value
Temperature	298 K
Volume of the adsorbate	100 ml
pH	7.0
Concentration of an adsorbent material	90 mg/dm^3^

The adsorbate used in the study was phenol. To the volume of it (see Table 3), an adsorbent material with the constant concentration (see Table 3) was added. Such prepared sample was shaken for 60 min. Before marking, samples were filtered through a membrane with a pore size of 0.45 μm, after which the removal of adsorbent material was achieved. Equilibrium results can be analyzed using the Freundlich or Langmuir adsorption isotherm method. In previous studies, it was realized that the degree of matching the theoretical Freundlich adsorption isotherm to experimental data is better than the Langmuir isotherm method (Dudziak and Werle 2016).

The Freundlich model is given as:

1 $$ {\mathrm{q}}_{\mathrm{eq}}={\mathrm{K}}_{\mathrm{f}}\times {\mathrm{C}}_{\mathrm{eq}}^{\raisebox{1ex}{$1$}\!\left/ \!\raisebox{-1ex}{$\mathrm{n}$}\right.} $$

where

*q*_eq_ is the quantity of the adsorbate per specified amount of adsorbent (mg/g),

*C*_eq_ is the equilibrium strength in solution (mg/dm^3^), and

*K*_f_ and *n are* the Freundlich constants.

This is an empirical equation, based on sorption on a heterogeneous surface, which can be presented as a linear function. This form makes it possible to identify the constants *K*_f_ and *n*:

2 $$ \log\ {q}_{\mathrm{eq}}=\log\ {K}_{\mathrm{f}}+\frac{1}{n}\log\ {C}_{\mathrm{eq}} $$

## Results and discussion

### Ultimate and proximate analysis and occurrences of organic and inorganic contaminants

The proximate and ultimate analyses of both SS1 and SS2 are displayed in Table 4. The ultimate analysis was carried out using the infrared spectroscopy analyzer. The following procedures and standards were used for SS characterization. Moisture content was determined according to EN ISO 18134-3:2015. The standard PN-EN 15402:2011 was adopted to determine the volatile content and the PN-EN 15403:2011 standard for the ash content. The CEN/TS15400:2006 procedure was used for the calorific value determination (HHV–the higher heating value).

**Table 4:** Proximate and ultimate analysis of investigated samples, % mass; average values (Werle and Dudziak 2015)

Parameters	SS1	SS2
M–Moisture	5.30	5.30
A–Ash	49.00	51.50
VM–Volatile matter	44.20	36.50
Carbon	27.72	31.79
Hydrogen	3.81	4.36
Oxygen	3.59	4.88
Nitrogen	13.53	15.27
Sulphur	1.81	1.67
Flour	0.003	0.013
Chloride	0.033	0.022
HHV, kJ/kg(dm)	1171	1405

### Gasification experiments results

During the experiment, information about the impact of the air ratio on combustible element content in gas, LHV of gas, and temperature distribution was acquired.

**Fig. 2:**
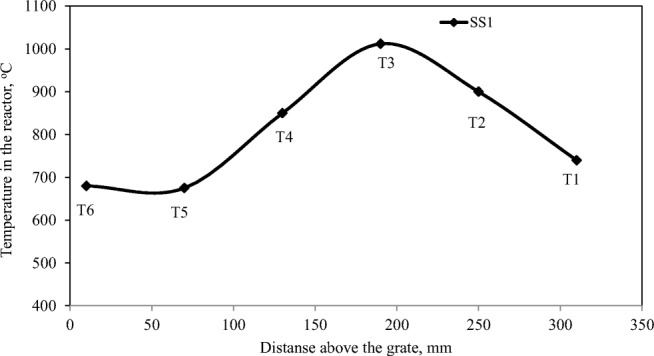
The distribution of the temperature; SS1 gasification

The temperature distribution in the reactor was obtained by measuring the temperatures at six characteristic points (*T*_1_ to *T*_6_) of the reactor (Fig. 2). The point *T*_1_ was located at 10 mm above of the gate. The next points were located 50 mm higher. The last point, *T*_6_, was located at 260 mm above of the grate.

Visual inspection of Fig. 2 indicates that measuring point *T*_3_ had the highest temperature values. This is due to the location of *T*_3_ in the oxidation zone. This zone is always the hottest area in the FBGs. Similarly, the measuring points *T*_6_ and *T*_5_ may be located in the drying zone, *T*_4_ in the pyrolysis zone, *T*_2_ in the oxidation (combustion) zone, and *T*_1_ in the ash zone. A similar pattern was observed during the experiment with the SS2 sample. The temperature pattern presented here is almost identical to other FBG reactors investigated in other gasification studies (Kim et al. 2016; Vonk et al. 2019).

**Fig. 3:**
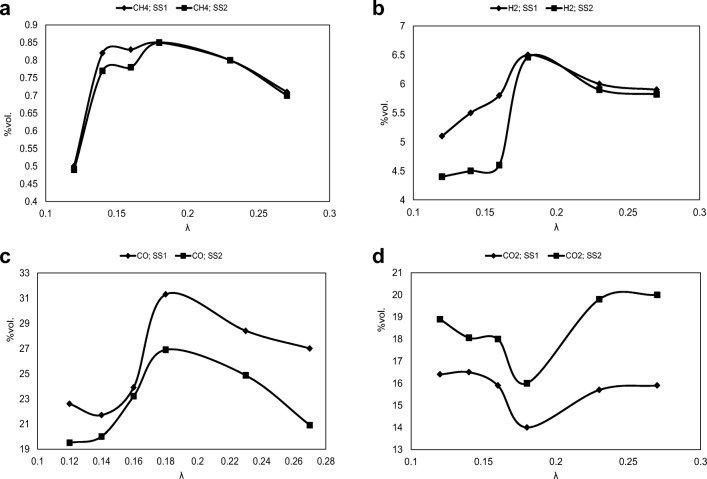
The volume fraction of main components in gas from SS1 and SS2 gasification process—impact of the air ratio. **a** CH_4_. **b** H_2_. **c** CO. **d** CO_2_

The impact of the air ratios on the main species content in the gasification gas for both analyzed samples is presented in Fig. 3.

The data presented in Fig. 3 show that in the whole range of *λ*, the volume fractions of carbon monoxide and hydrogen are higher for SS1 than for SS2. In terms of lower values of *λ*, the carbon monoxide fraction was found to be low. The highest value of this fraction (31.3% for SS1 and 26.9% for SS2) is observed for *λ* equal to 0.18. Similar results were achieved in other studies. Kim et al. (2016) used a FBG for SS gasification briquettes. The highest achieved volume fraction of CO was equal to 32.6%.

A sudden increase in the CO amount of this value is due to the main role of the primary water-gas reaction. After exceeding this value, a decrease of CO fraction was observed. Analysis of the course of carbon dioxide fraction changes shows an inverse relationship with carbon monoxide. This is due to the participation of these compounds in identical chemical reactions—thus, the amount of CO_2_ is the smallest when the fraction of CO is the largest.

The chemical reactions taking place in the gasification reactor are the result of the reaction of the gasification agent with the fuel and the same factor with the gas phase CO.

The gasification agent reacts with carbon in endothermic reactions, mainly producing CO (via CO + H_2_O → CO + H_2_). On the other hand, the gasification agent reacts exothermic with CO, producing mainly carbon dioxide and hydrogen—via CO + H_2_O↔CO_2_ + H_2_. This is the main reason for the observed change in gas composition as a function of *λ* changes. Similar gas composition has been observed during the gasification experiments of SS in air (Kim et al. 2016). In their study, a FBG reactor produced gas with combustible components of CO, H_2_, and CH_4_ with concentrations of 10%, 5%, and < 1% respectively. This small amount of methane was also confirmed by, for example, Ayol et al. (2019), in which SS FBG results were presented, with the volume fraction of methane equal to 1.2%.

**Fig. 4.**
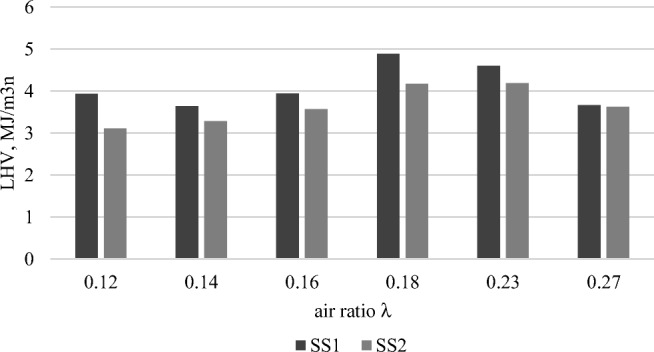
The LHV of gasification gas from gasification of the SS1 and SS2 as a function of the air ratio

The impact of the *λ* on the LHV of gasification gas is presented in Fig. 4. The following formula from Kim et al. (2011) was used:

3 $$ \mathrm{LHV}=0.126\cdotp \mathrm{CO}+0.108\cdotp {\mathrm{H}}_2+0.358\cdotp {\mathrm{CH}}_4 $$

Figure 4 shows LHV reaches its maximum at an air ratio value of *λ* = 0.18. This was the case for both sewage samples. Above that value of *λ*, the thermochemical process could result in a shift from gasification to combustion.

**Fig. 5.**
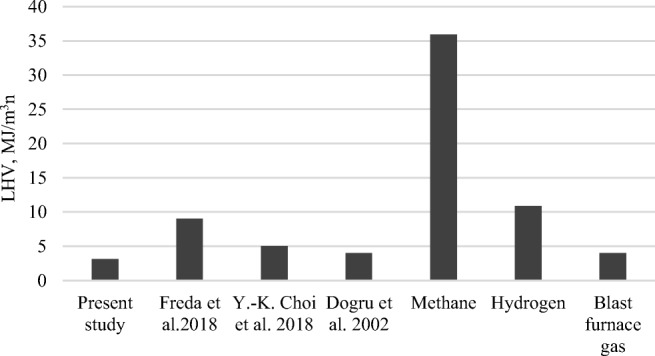
The LHV of gasification gas from gasification (medium value) in comparison to other gasification gases from previous studies (2002-2018) and other gaseous fuels

Gasification results show that process gas is characterized by the LHV in comparison with popular gaseous fuels, up to 5 MJ/m^3^_n_ (see Fig. 5).

While the LHV of gasification gas is much lower than methane or hydrogen, it is still comparable with the LHV of blast furnace gas. The present data indicates that the gasification gas can be used as a fuel in power engineering systems. Chemistry purposes are also possible (e.g., synthesis process).

Freda et al. (2018) showed gasification experiment results, carried out in a bench scale rotary kiln under simulated autothermal condition. Dry gas having a high heating value of 6–9 MJ/m^3^_n_ and a tar content of 4–6 g/m^3^_n_ was obtained at a temperature of 800–850 °C, with an air ratio between 0.15 and 0.24. Dogru et al. (2002) showed SS gasification experiments using a 5 kWe-throated downdraft gasifier. The low-quality combustible gas was produced. It is acceptable as a fossil fuel replacement, with an LHV of 4 MJ/m^3^_n_ that would still allow an internal combustion engine to operate. Air gasification of dried SS was performed in a FlBG, resulting in a mean value of 5 MJ/m^3^_n_ and information about application of tar-removal from biomass gasification (Choi et al. 2018).

All of the studies discussed above show that despite temperature range, the mean value of gasification gas depends strongly on air ratio, the optimal value of which, adequate to reactor technology, yields the highest heating values of the gas.

### Phosphorus recovery potential

One of the important targets of the CE approach is nutrient extraction from waste materials. One such waste material is SS. Despite the visible progress in methods of WWT, there are no optimal results in phosphorus recovery from the wastewater stream. Additionally, some commercial coagulants used in those technologies create difficulties in potential recovery of P from SS. Sorption materials may be a good solution for WWT.

As was mentioned above, the main feature of the gasification technology, due to reductive atmosphere during the process, is that inorganic compounds are moved into the solid phase. So, it can be postulated that gasification of SS is the most effective solution taking into consideration the potential recovery of phosphorus. Present results show that the solid fraction after gasification is a promising source of phosphorus in comparison to other residues (e.g., ashes) from SS combustion. Nevertheless, both types of residues are characterized by different chemical and technological parameters than natural sources of phosphorus (e.g., phosphate ore). Due to this, such alternative material should be deeply analyzed including the possibility of its the addition to standard material.

The solid fraction of the SS gasification process consists of 20.06% P_2_O_5_ (Gorazda et al. 2018). This value is comparable to ash from the SS combustion process (22.47% of P_2_O_5_). However, Gorazda et al. (2017, 2018) show that the content of microelements, such as Fe, Cu, Zn, and Mn, have differed from those of natural source of phosphorus. These micronutrients are essential for plant growth, and their values have to be controlled for the production of fluid fertilizers, in accordance with Regulation EC no 2003,2003. Wilfert et al. (2018) stated that phosphate recovery from SS is essential to the prospect of a CE, as it is one of the most feasible ideas and has been the subject of numerous studies (Viader et al. 2015; Gorazda et al. 2018). Acelas et al. (2014) investigated the possibility of gaseous fuel production along with P extraction using the SCWG (supercritical water gasification) method. After SCWG, majority (> 95%) of the phosphorus could be recovered.

Gorazda et al. (2017) leached solid phase with phosphoric and nitric acids, according to patented PolFerAsh technology. It was concluded that the efficiency of the phosphorus leaching from solid fraction after gasification is higher than 73%. What is more, most of the iron and heavy metals remain in the solid residue due to the low concentration of acids and the specific solid to liquid phase ratio. Viader et al. (2015) investigated low-temperature gasification of SS with emphasis on P extraction from gasification ash. The study concluded that using a 2-compartment electro dialytic (ED) setup for P separation, it was possible to extract up to 26% of the P from pure SS ashes, while up to 90% of the P was extracted from the ashes from the gasification of SS and straw pellets mixture.

### Adsorption process efficiency

The very important feature of the CE concept, which distinguishes it from a linear, industrial model is flexibility and not being subject to general schemes. In this context, the CE is looking for new, unconventional materials that will be used in industry and engineering. Flexibility is also related to the new, previously unrealized, use of a given (not necessarily new) material. Such materials go beyond the general scheme and must be tested before being used. The adsorption process of the various types of contaminant on unconventional adsorbent materials is an example of such activity. During adsorption, the molecules of a substance from a gas mixture (or liquid solution) become attached to a solid (or liquid) surface.

**Fig. 6:**
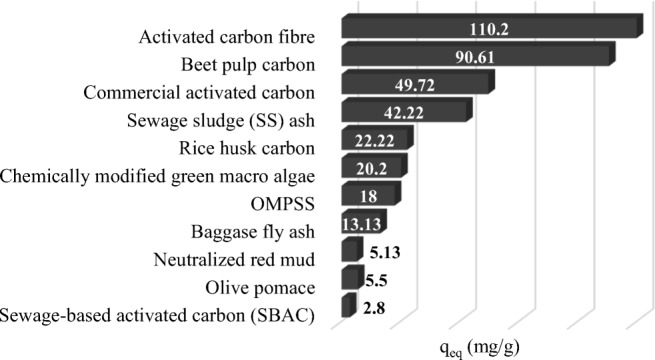
Adsorption capacity of organic compounds—comparison of SS gasification ash with other sorbents

It is demonstrated in Fig. 6 that solid gasification by-products (SS ash) can be used as an adsorption material in order to eliminate organic contaminants from water streams, (e.g., phenols). In this figure is presented the comparison maximum of the adsorption capacity of the phenol monolayer on different adsorbents. Taking this result into consideration, it can be assumed that the efficiency of phenol adsorption on the SS gasification ash was higher than for other unconventional adsorbents (e. g., chemically modified green macro algae). Björklund and Li (2017) investigated the adsorption of hydrophobic organic compounds usually detected in stormwater. Sludge-based activated carbon (SBAC) was as efficient as tested commercial carbons for adsorbing organic compounds (up to 2.8 mg/g). SS as an activated carbon source has great potential as a renewable alternative for SS WM practices and for the production of an effective adsorption material. Zhang et al. (2018) propose the one-step microwave pyrolysis of sewage sludge (OMPSS) as a process to produce the unconventional adsorbents for organic contaminants. The average value of the adsorbed substance per amount of adsorbent was equal to 18 mg/g.

The results presented in this chapter show that gasification is a very useful method of sewage sludge management. The advantages of gasification are particularly evident, when we think about the CE idea. The reductive nature of the process means that many valuable products, e.g., phosphorus, are moved to a solid phase. In addition, the specificity of the process leads to the formation of a solid waste products which can be used as an unconventional adsorbent. The advantage associated with the production of combustible gas, which is a source of energy in technical devices, such as boilers, engines, or gas turbines, is indisputable. In Poland, the sewage sludge gasification is still a perspective technology. Although, Poland ranks fifth place behind Czech Republic, Greece, The Netherlands, and Germany taking into account number of incentives for biomass (Banja et al. 2019) in heat and electricity production sector, the similar incentive tools for waste utilization are rather rare. We need to remove the regulatory barrier for sewage sludge deployment and create more financial, administrative and formal incentives. In Poland, there is an impressive experience in biomass gasification (Werle 2015). Therefore, it is optimistic to look at the development of this technology also in the context of SS. The CE idea introduced into EU law can helps this. The installation by Zamer of a fluidized bed EKOD gasifier (Stelmach and Wasilewski 2012), ICHPW of a fixed bed GazEla gasifier (Iluk et al. 2016), or installation presented in this paper are good examples of this perspective.

## Conclusions

Within the frame of this paper, the municipal SS gasification as an element of the CE concept has been presented. It has been denoted, that air ratio and temperature in the analyzed process had great impact on the process gas composition. The volume fraction of the combustible gasification gas elements (carbon monoxide, hydrogen, and methane) were increased with higher temperature and oxygen content in the gasifying agent. Higher values of the carbon and hydrogen in gasified fuel (SS1 and SS2) affect the increase of the LHV of gasification gas. It reached the highest value for *λ* = 0.18. The LHV of gasification gas is comparable with the LHV of blast furnace gas. Unfortunately, the LHV is much lower in comparison to popular gaseous fuel, like methane. Solid waste by-products from SS (ashes) can be used as an adsorbent for the elimination of toxic organic substances from water streams (e.g., phenols). The received adsorbent from SS gasification should be subjected to deep purification processes. The efficiency of phenol adsorption from water solution on SS gasification solid product was higher than for other analyzed adsorbents, like rice husk carbon or olive pomace. The post-process gasification solid fraction is a perspective source of P (20.06% P_2_O_5_). It is almost as high as for SS ash (22.47% P_2_O_5_) and natural phosphate rocks (28.05% P_2_O_5_). The chemical properties and technological parameters differ from natural phosphate sources. Thus, such material should be well recognized and treated separately. The results of this analysis indicate that there is great potential for improvement in sludge management utilizing the CE perspective. These results and conclusions ought to be presented to authorities and water treatment plant management in order to propose an increase in energy efficiency and profitability of SS production and utilization. Examples of the first solutions in the technology of gasification of SS in Poland may be an argument in the discussion of create directions of management of this group of raw materials.
